# Preparation of a Nanoemulsion with* Carapa guianensis* Aublet (Meliaceae) Oil by a Low-Energy/Solvent-Free Method and Evaluation of Its Preliminary Residual Larvicidal Activity

**DOI:** 10.1155/2017/6756793

**Published:** 2017-07-17

**Authors:** Flávia L. M. Jesus, Fernanda B. de Almeida, Jonatas L. Duarte, Anna E. M. F. M. Oliveira, Rodrigo A. S. Cruz, Raimundo N. P. Souto, Ricardo M. A. Ferreira, Regina Gendzelevski Kelmann, José C. T. Carvalho, Ana C. Lira-Guedes, Marcelino Guedes, Conxita Solans, Caio P. Fernandes

**Affiliations:** ^1^Post-Graduate Program in Tropical Biodiversity, Federal University of Amapá, Rodovia Juscelino Kubitschek, KM-02, Macapá, AP, Brazil; ^2^Laboratory of Phytopharmaceutical Nanobiotechnology, Federal University of Amapá, Rodovia Juscelino Kubitschek KM-02, Macapá, AP, Brazil; ^3^Laboratory of Drug Research, Federal University of Amapá, Rodovia Juscelino Kubitschek, KM-02, Macapá, AP, Brazil; ^4^Laboratory of Arthropoda, Federal University of Amapá, Rodovia Juscelino Kubitschek, KM-02, Macapá, AP, Brazil; ^5^Faculdade de Farmácia e Bioquímica, Campus Governador Valadares, Universidade Federal de Juiz de Fora, Juiz de Fora, MG, Brazil; ^6^Brazilian Agricultural Research Corporation (EMBRAPA), Rodovia Juscelino Kubitschek, No. 2600, Macapá, AP, Brazil; ^7^Institute of Advanced Chemistry of Catalonia (IQAC-CSIC), C/Jordi Girona, No. 18-26, Barcelona, Spain

## Abstract

Andiroba (*Carapa guianensis*) seeds are the source of an oil with a wide range of biological activities and ethnopharmacological uses. However, few studies have devoted attention to innovative formulations, including nanoemulsions. The present study aimed to obtain a colloidal system with the andiroba oil using a low-energy and organic-solvent-free method. Moreover, the preliminary residual larvicidal activity of the nanoemulsion against* Aedes aegypti* was evaluated. Oleic and palmitic acids were the major fatty acids, in addition to the phytosterol *β*-sitosterol and limonoids (tetranortriterpenoids). The required hydrophile-lipophile was around 11.0 and the optimal nanoemulsion was obtained using polysorbate 85. The particle size distribution suggested the presence of small droplets (mean diameter around 150 nm) and low polydispersity index (around 0.150). The effect of temperature on particle size distribution revealed that no major droplet size increase occurred. The preliminary residual larvicidal assay suggested that the mortality increased as a function of time. The present study allowed achievement of a potential bioactive oil in water nanoemulsion that may be a promising controlled release system. Moreover, the ecofriendly approach involved in the preparation associated with the great bioactive potential of* C. guianensis* makes this nanoemulsion very promising for valorization of this Amazon raw material.

## 1. Introduction

Various nontimber products are subject of sustainable use in the Amazon region, including* Carapa guianensis* Aublet (Meliaceae) [1].* C. guianensis *is commonly known as andiroba but can also be known as karapa, krapa, or crabwood, among other popular names. The oil extracted from andiroba seeds has many industrial applications in pharmaceuticals and cosmetics. In addition, the residue from oil production is used for the preparation of insect repellent candles [2]. Ethnopharmacological uses of this oil include the treatment of arthritic pain, ear infections, and skin diseases, including psoriasis, and being a topical natural insect repellent [3].

Antiallergic and antihyperalgesic effects of andiroba oil have been found to be associated with limonoids (tetranorterpenoids), which are considered characteristic phytochemicals of this species [4]. These substances are also related to anti-inflammatory effects of* C. guianensis* [5]. The antiplasmodial in vitro activity of andiroba oil and absence of toxic effects in mice supported the antimalarial traditional use of this Amazon raw material [6]. Although its acute and subacute administration did not exhibit toxic effects in male Wistar rats and hematological parameters were not altered, changes in some biochemical parameters might be associated with hepatic injury [7]. This oil did not induce toxic effects in female pregnant Wistar rats, suggesting a possible safety during pregnancy [8]. Another remarkable potential of andiroba oil is associated with its larvicidal activity against* Aedes aegypti *[9, 10], the main vector of dengue, and several other tropical neglected or emerging diseases. The wide range of biological activities and ethnopharmacological properties makes andiroba oil a valuable raw material for different industrial sectors, predominantly pharmaceutical, cosmetic, and pesticide industries.

Some pharmaceuticals have been proposed using andiroba oil, such as polymeric microparticles [11], emulsions [12], emulsions with liquid crystals [13], and even oil in water nanoemulsions [14]. Particular attention has been observed in recent years to the preparation of oil in water nanoemulsions from natural products [15]. They are transparent or translucent dispersed systems of fine oil droplets in aqueous continuous phase. Despite the fact that nanoemulsions are thermodynamically instable, they differ from conventional emulsions since they can reach kinetic stability, being more resistant against sedimentation and creaming [16].

To our knowledge, few efforts have been performed to obtain a bioactive nanoemulsion from andiroba oil. Thus, the focus of the present study is to generate this system using a low-energy and organic-solvent-free method, considering that this can be considered an ecofriendly approach. Moreover, we evaluated the preliminary residual larvicidal activity of the nanoemulsion against* Aedes aegypti*, considering (i) the fact that andiroba oil is active against this vector larvae and (ii) the simplicity of the bioassay to suggest that the nanoemulsion is active and has potential applications for previous biological activities related to the oil.

## 2. Materials and Methods

### 2.1. Plant Material

Andiroba (*C. guianensis*) seeds (registration number 192790) were obtained from the Brazilian Agricultural Research Corporation (Embrapa Amapá) of the state of Amapá. They were collected at a várzea environment in the municipality of Mazagão (WGS 84, Zone 22 N, 469483,911 L, and 9987772,006 N). The plant material was dried in an oven at constant temperature (45°C) until constant weight and the oil was obtained by cold pressing using a MA098/C hydraulic press.

### 2.2. Gas-Chromatograph Analysis

Prior to gas-chromatograph analysis, the andiroba oil was fractionated using silica gel as stationary phase in order to facilitate detection of the limonoids. Elution was performed with hexane (200 mL), hexane : ethyl acetate (1 : 1; 200 mL), and ethyl acetate (200 mL). The ethyl acetate fraction was diluted in dichloromethane and analyzed using a GCMS-QP5000 (SHIMADZU) gas chromatograph equipped with a mass spectrometer, using electron ionization, according to the following experimental conditions: injector temperature, 270°C; detector temperature, 290°C; carrier gas, helium; flow rate, 1 mL/min; split injection with split ratio 1 : 5. The oven temperature was programmed from 60°C (isothermal phase for 3 min), with an increase of 10°C/min to 290°C, ending with a 30 min isothermal phase at 290°C. ZB-5MS column had the following characteristics: i.d. = 0.25 mm, length 30 m, film thickness = 0.25 *µ*m. Mass spectrometry (MS) conditions were as follows: ionization voltage, 70 eV; scan rate, 1 scan/s; mass range,* m/z *30–600. Identification of the compounds was performed by comparison of the MS fragmentation pattern of each substance with data available in the literature.

### 2.3. Nanoemulsions

#### 2.3.1. Determination of Required Hydrophile-Lipophile Balance (rHLB) of Andiroba Oil

The nonionic surfactants polysorbate 80 and sorbitan monooleate were blended in order to achieve a wide range of hydrophile-lipophile balance (HLB) values as follows: 4.3 (100% of sorbitan monooleate), 5, 6, 7, 8, 9, 10, 11, 12, 13, 14, and 15.0 (100% of polysorbate 80). All components were pooled together in a screw top vial and vigorously mixed using a vortex stirrer. 90% (w/w) of water, 5% (w/w) of surfactant (s), and 5% (w/w) of* C. guianensis* oil, at a final mass of 5 g, constituted each emulsion. Macroscopical evaluation was performed immediately after preparation and after 7 days of storage at room temperature (25 ± 2°C).

#### 2.3.2. Evaluation of Influence of Surfactants on Nanoemulsion Formation

Influence of different surfactants on nanoemulsion formation was evaluated. Single surfactants or blends close to rHLB value were used as follows: polysorbate 81 (HLB = 10), polysorbate 85 (HLB 11), polysorbate 80/polysorbate 85 (HLB = 11.5), polysorbate 20/polysorbate 81 (HLB = 12), polysorbate 20/polysorbate 85 (HLB = 12), polysorbate 80/polysorbate 81 (HLB = 12), polysorbate 80/polysorbate 85 (HLB = 12), and polysorbate 80/polysorbate 85 (HLB = 12.5). The system was vigorously mixed using a vortex stirrer and water was added drop by drop. Andiroba oil to surfactant ratio was 1 : 9 and water final content was 95% (w/w). Macroscopical evaluation was performed immediately after preparation and after 1 day of storage at room temperature (25 ± 2°C). Nanoemulsions were reported when translucent or transparent aspect together with a bluish reflect was observed and macroscopical evaluation was used as criteria for choice of optimal andiroba nanoemulsion.

#### 2.3.3. Physical Characterization of the Optimal Andiroba Nanoemulsion

The optimal andiroba nanoemulsion was characterized using a Zetasizer Nano ZS. It was diluted in deionized water (1 : 25, v/v) and droplet size, polydispersity index, and zeta potential were recorded. Each measurement was performed in triplicate and data were expressed as the mean ± standard deviation. The nanoemulsions were also subjected to analysis using a programmed linear ramp of temperature starting from 25°C to 80°C and measurements were performed at 10°C intervals. Results are expressed as mean ± standard deviation.

### 2.4. Preliminary Residual Larvicidal Assay

The preliminary residual larvicidal activity was carried out according to Kanis et al. [17], with some modifications. Late third instar/early fourth instar larvae of* Aedes aegypti* (Rockefeller strain) were obtained from the Arthropod Laboratory (Federal University of Amapá, Brazil) and experimental conditions were as follows: water temperature, 25 ± 2°C; relative humidity of the room, 75 ± 5%; and a 12 h dark : light controlled ambient. All experiments were performed in triplicate with 10 larvae in each replicate. Assays were carried out in glass beakers and the final volume of aqueous media in each replicate was 100 mL. The treated group beaker contained the optimal nanoemulsion diluted at 250 ppm (expressed as andiroba oil content in aqueous media). Mortality was recorded after 24 h of treatment and, then, all larvae were removed. The aqueous media containing nanoemulsion were left in the beaker for an additional period of 24 h, corresponding to one cycle of 48 h. Then, a filtration step using qualitative Whatman paper was performed. New larvae were added to the filtrate, initiating a new cycle of 48 h. In all, three cycles of 48 h were performed, comprising three sets of larvae addition and constituting the entire preliminary residual larvicidal assay. The control group was subjected to the same procedure, using only water. Analysis of variance (two-way ANOVA) followed by Tukey's test or Bonferroni's test was conducted using the Software GraphPad Prism 6.0 (San Diego, California, USA). Differences were considered significant when *p* ≤ 0.05.

## 3. Results and Discussion

**Figure 1:**
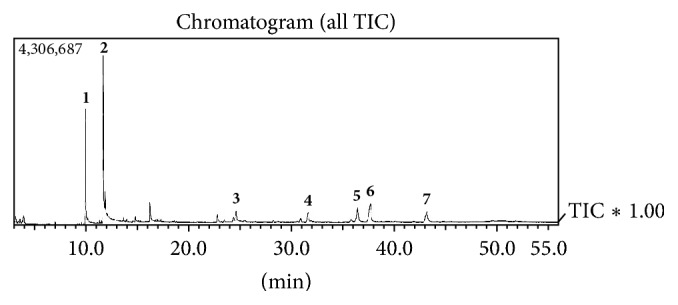
Total ion chromatogram after analysis of* C. guianensis *oil by gas-chromatography coupled to a mass spectrometer.

**Figure 2:**
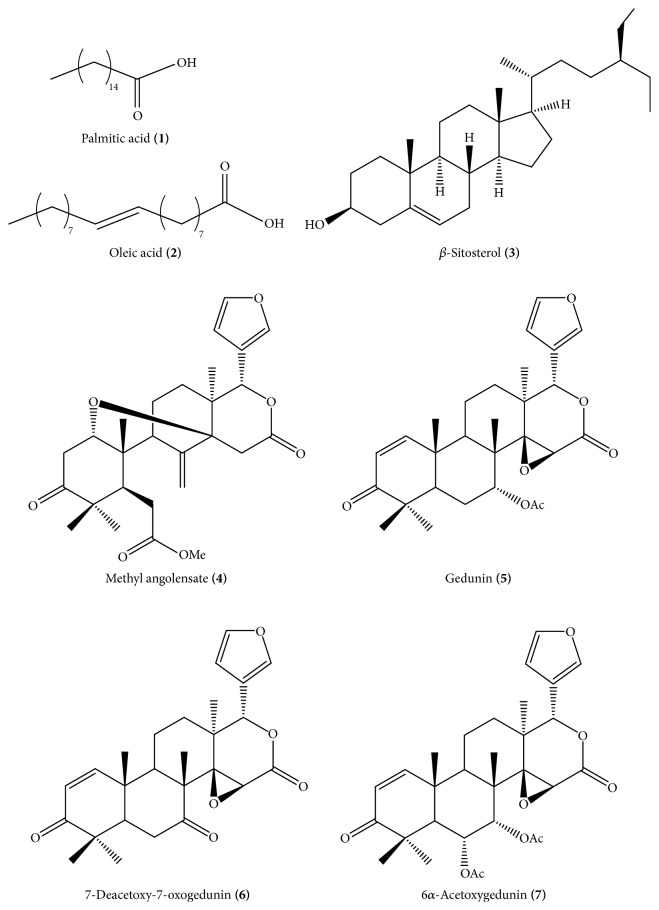


Figure 1 shows the total ion chromatogram after analysis of* C. guianensis *oil by gas-chromatography coupled to a mass spectrometer. Substances with retention times of 10.01 min and 11.73 min presented molecular ion peaks (M^+^) at* m/z *256 and 282, in accordance with palmitic acid (**1**) and oleic acid (**2**), respectively. These compounds have been previously reported as major constituents of* C. guianensis *seed oil [18, 19]. The substance with a retention time of 24.67 min presented a molecular ion peak at (M^+^)* m/z *414 and also a peak at* m/z* 396. These fragments are in accordance with literature data of *β*-sitosterol [20, 21], suggesting the presence of this phytosterol in the oil. The substance with a retention time of 31.69 min presented a molecular ion peak at* m/z *470, in accordance with methyl angolensate (**4**). The substance with a retention time of 36.49 min presented a high abundant peak at* m/z* 299. The absence of a peak at* m/z *317 suggested that this compound is gedunin (**5**). The substance with a retention time of 37.69 min presented the base peak at* m/z* 315, and a peak at* m/z* 438 that is attributable to a molecular ion peak (M^+^) was also observed, suggesting that this compound is 7-deacetoxy-7-oxogedunin (**6**). The substance with a retention time of 43.21 min presented a high abundant peak at* m/z* 297, suggesting that this compound is 6*α*-acetoxygedunin (**7**). Comparison of mass spectra data of these compounds with literature data [22] allowed identification of the limonoids** 4**–**7**. Chemical structures of substances identified in the* C. guianensis *oil are presented in Figure 2.

**Figure 3:**
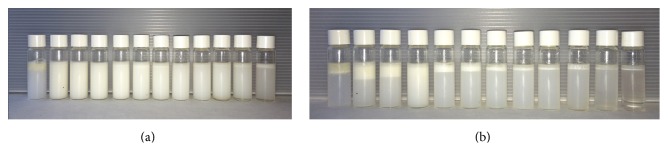
Set of emulsions prepared with* C. guianensis* oil and sorbitan monooleate/polysorbate 80 at different ratios (from left to right: HLB 4.3; 5; 6; 7; 8; 9; 10; 11; 12; 13; 14; and 15). (a) represents macroscopical characteristic of the emulsions on the day of preparation. (b) represents macroscopical characteristic of the emulsions after 7 days of storage (25°C).

Figure 3 shows the set of emulsions that were prepared with andiroba oil and different blends of sorbitan monooleate/polysorbate 80 at a wide range of hydrophile-lipophile balance (HLB). Those prepared solely with sorbitan monooleate or polysorbate 80, as well as the emulsions prepared with surfactant blend at HLB of 14, presented instable behavior immediately after preparation. Remaining emulsions prepared with surfactant blends at HLB 5–13 presented an opaque aspect with some degree of creaming. After 7 days of storage, less alteration and lower creaming were observed for emulsions at HLB 11 and 12, followed by emulsion at HLB of 10. The concept of required HLB (rHLB) is based on the fact that the most stable emulsion is formed when the HLB of the surfactant (s) coincides with the rHLB of the oil. Therefore, the preparation of a set of emulsions with a pair of nonionic surfactants at different ratios, as well as subsequent evaluation of the stability of each emulsion, is a valuable tool to identify this parameter and even achieve small droplets, including nanoemulsions [23, 24]. Ferreira et al. [12] performed rHLB determination of andiroba oil using a high-speed homogenizer and 20 sorbitan monooleate/polysorbate 20 blends. It was concluded that the rHLB of the oil was 16.7, indicating that the best emulsion was achieved solely with polysorbate 20. However, these authors also suggested that the size of the droplets was independent of the HLB value of the surfactant(s) and long-term stability study (120 days) showed even lower creaming index for the formulation obtained with surfactants at HLB 11.2 (CI % = 1.47), when compared to formulation obtained at HB 16.7 (CI % = 2.98). It is well established that the film formed by nonionic surfactants around the droplets develops a main role in the stabilization of the disperse system [25]. This is highlighted by the fact that using different single surfactants or surfactant pair at a fixed HLB value and differences in the mean droplet size and stability may occur [26]. Considering that we used a different hydrophilic surfactant in the required HLB value determination, this fact should also be considered. Thus, based on the aforementioned discussion, we suggest that the rHLB of this oil is around 11.

The difference in the required HLB value may also be associated with qualitative and/or quantitative variation in phytochemicals of the oils or some degree of degradation during storage. Further studies using andiroba oils from different origins, freshly extracted and stored for long periods, should be encouraged in this issue, since vegetable oils are more complex and less deeply studied in colloidal systems. Emulsions containing individual vegetable oils, including andiroba, as internal phase were also prepared, independent of the rHLB value [13].

**Figure 4:**
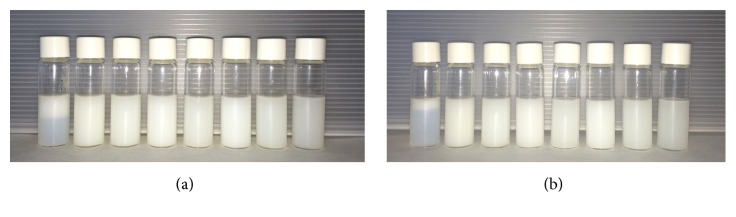
Set of emulsions prepared with* C. guianensis *oil and single surfactants or blends as follows (from left to right): polysorbate 81 (HLB = 10), polysorbate 85 (HLB 11), polysorbate 80/polysorbate 85 (HLB = 11.5), polysorbate 20/polysorbate 81 (HLB = 12), polysorbate 20/polysorbate 85 (HLB = 12), polysorbate 80/polysorbate 81 (HLB = 12), polysorbate 80/polysorbate 85 (HLB = 12), and polysorbate 80/polysorbate 85 (HLB = 12.5). (a) represents macroscopical characteristic of the emulsions on the day of preparation. (b) represents macroscopical characteristic of the emulsions after 7 days of storage (25°C).

Aiming to obtain an optimal nanoemulsion for bioactivity evaluation, we investigated the influence of different surfactants on nanoemulsions prepared with andiroba oil.  Figure 4 shows that only the formulation prepared with polysorbate 81 (HLB = 10) presented any obvious sign of instability, while the other formulations presented opaque to slightly translucent aspect and bluish reflect, suggesting formation of nanoemulsions. The classical approach for evaluation of influence of surfactant type involves utilization of nonionic surfactants. Often, the surfactant pair is composed of a low HLB surfactant and a high HLB surfactant [25] instead of single surfactants. It is worth mentioning that natural oils with dissolved secondary metabolites are intrinsically different from classical fatty acid oils or synthetic oils. Although the nanoemulsions obtained with polysorbate 80/polysorbate 85 (HLB = 11.5), polysorbate 20/polysorbate 81 (HLB = 12), polysorbate 20/polysorbate 85 (HLB = 12), polysorbate 80/polysorbate 81 (HLB = 12), polysorbate 80/polysorbate 85 (HLB = 12), and polysorbate 80/polysorbate 85 (HLB = 12.5) presented potential for generating andiroba oil nanoemulsions, we opted for the nanoemulsion prepared with polysorbate 85 as the optimal nanoemulsion. This surfactant was also considered very promising for another oil in water nanoemulsion prepared with complex Amazon oils (data not shown) and reaches the rHLB value that we determined in the present study.

**Figure 5:**
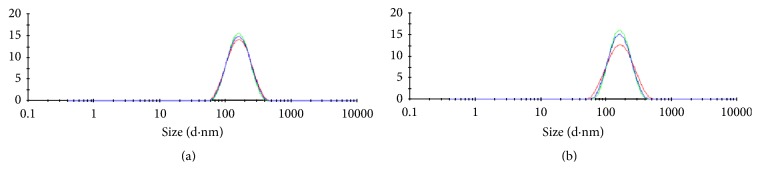
Particle size distribution of oil in water optimal nanoemulsion prepared with* C. guianensis *oil. Day 0: mean droplet size = 148.8 ± 0.2646 nm and polydispersity index = 0.131 ± 0.012. Day 1: mean droplet size = 151.5 ± 0.6028 and polydispersity index = 0.132 ± 0.017.* C. guianensis* oil content in the nanoemulsion was 5000 ppm.

**Figure 6:**
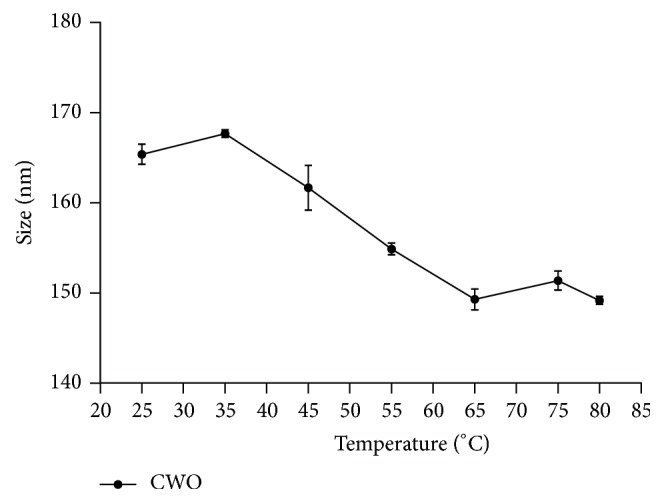
Influence of temperature on droplet size of oil in water optimal nanoemulsion prepared with* C. guianensis *oil. Each measurement represents mean ± standard deviation.

**Figure 7:**
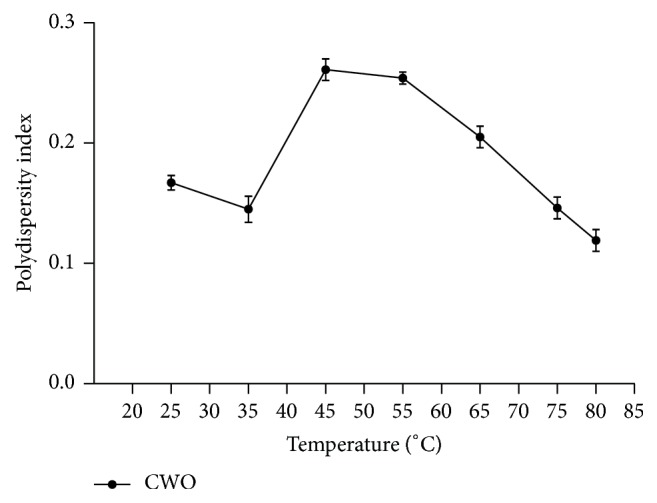
Influence of temperature on polydispersity index of oil in water optimal nanoemulsion prepared with* C. guianensis *oil. Each measurement represents mean ± standard deviation.

Dynamic light scattering analysis (Figure 5) revealed that the optimal nanoemulsion prepared with andiroba oil and polysorbate 85 presented a small mean diameter (Day 0: 148.8 ± 0.2646 nm; Day 1: 151.5 ± 0.6028), corroborating its classification as a nanoemulsion. Low polydispersity indices (Day 0: 0.131 ± 0.012; Day 1: 0.132 ± 0.017) were also indicative of a narrow size distribution (Figure 5). Negative zeta potential values were also observed (Day 0: −24.2 ± 0.751 mV; Day 1: −27.6 ± 0.173 mV). In the literature, we found few examples of efforts to obtain nanoemulsions with andiroba oil, despite the knowledge that this Amazon raw material has a wide range of biological activities. Baldissera et al. [14] successfully generated andiroba oil-based nanoemulsions using a spontaneous emulsification approach, which involved utilization of acetone as a constituent of organic phase during the emulsification process. This nanoemulsion showed in vitro trypanocidal activity and presented a mean droplet size of around 240 nm and a polydispersity index of around 0.150. However, to our knowledge, the present study is the first to report preparation of nanoemulsions using* C. guianensis *oil by a low-energy and solvent-free method. Analysis of particle size distribution after temperature increase was also performed (Figures 6 and 7). The mean droplet size slightly increased, while the polydispersity index slightly decreased, in the range of 25 to 35°C. The mean droplet size decreased from 35 to 65°C in a linear manner, remaining almost constant from 65 to 80°C. The polydispersity index increased from 35 to 45°C, and then it slightly decreased from 45 to 55°C, presenting a tendency for a linear decrease from 55 to 80°C. It is well established that the flexibility of the interfacial film plays an integral role in droplet size. Thus, deposition and/or migration of compounds that influence this factor should be considered [25]. Considering the complexity of phytochemicals from* C. guianensis *oil, including some that may act as emulsifiers, rearrangement of droplets due to alteration in film flexibility may have occurred. Moreover, no major increase in droplet size accompanied by polydispersity index augmentation was observed, suggesting that Ostwald ripening did not trigger the changes observed in the present study. This is the main mechanism of nanoemulsion instability, in which substances released from the internal phase form bigger droplets [16]. However, deeper investigations on colloidal systems prepared with complex natural products (e.g., limonoids terpenoids, and flavonoids) should be performed for better understanding of mechanism involve.

**Figure 8:**
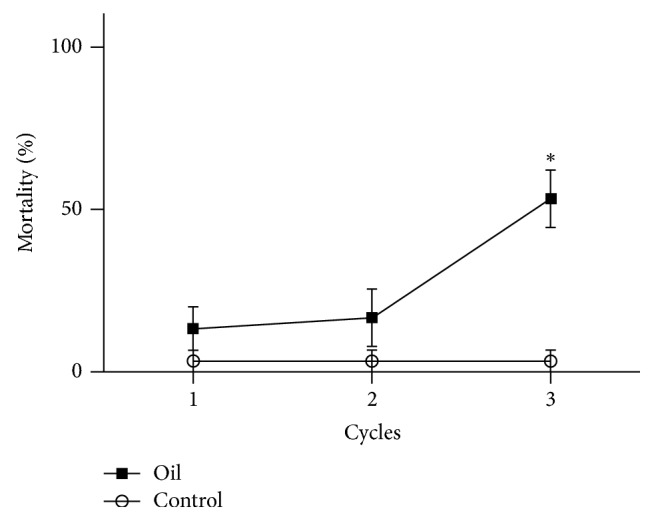
Residual larvicidal activity against* Aedes aegypti*. Oil, larvae treated with the optimal nanoemulsion prepared with* C. guianensis *oil at 250 ppm (expressed as a function of oil content in aqueous media). Control, untreated larvae. ^*∗*^Significant difference between treated and control groups (*p* < 0.001). Each cycle represents 48 h of experiment.

The optimal nanoemulsion was diluted to 250 ppm and evaluated against* Aedes aegypti *larvae. No statistically significant difference was observed between mortality levels of the treated and control groups after the first and second cycle, while a significant difference (*p* < 0.001) was observed after the third cycle (Figure 8). Mortality levels (3.33 ± 5.77%) in the control group were not dependent on cycles (*p* > 0.05). The mortality level reached in the treatment group after the third cycle (53.33 ± 15.30%) was considered statistically different (*p* < 0.01) from mortality levels after the first (13.33 ± 11.55%) and second (16.67 ± 15.30%) cycles.* A. aegypti *is the main vector of dengue, a tropical disease that affects millions of people worldwide. Moreover, nowadays, growing cases of emergent diseases, such as chikungunya fever and Zika, indicate that new agents for vector control are required.

Data in the literature suggest that the lethal concentration that kills 50% (LC50) of* A. aegypti *larvae (Rockefeller strain) after 24 h of treatment with andiroba oil ranges as a function of larval stage, such as first instar (LC50 = 48 ppm), second instar (LC50 = 126 ppm), third instar (LC50 = 106 ppm), and fourth instar (LC50 = 234 ppm) [9]. The lethal concentration has also been shown to be dependent on temperature, at 15°C (LC50 = 447 ppm), 20°C (LC50 = 386 ppm), 25°C (LC50 = 141 ppm), and 30°C (LC50 = 146 ppm) for late third instar/early fourth instar larvae [10]. Residual larvicidal assay indicated that maximum mortality was reached by the 12th day of treatment with* C. guianensis *oil at 1400 ppm and that it then gradually decreased [27]. In the present study, we observed that mortality increased as a function of time. This can be explained by the fact that, in the beginning of the experiment, most of bioactive compounds are entrapped in the nanoemulsions. As they are gradually released, the sum of concentration of bioactive compounds that were initially available at the aqueous media and additional released compounds may be responsible for increased larvicidal activity. This is the opposite of conventional profile expected for non-nanoemulsified systems, in which the biological activity (e.g., mortality of larvae) decreases as a function of time due to degradation of bioactive compounds. However, further studies aiming to quantify the chemical markers of andiroba oil and correlate this data with mortality levels evaluated according to WHO phase II (small-scale field trials) and phase III (large scale field trials) studies [28] should be performed to validate this hypothesis and ensure that an undoubted residual activity is associated with a controlled release of compounds. Moreover, evaluation of these further assays at different days in order to ensure the reproducibility of the results in the field should be highlighted in order to provide experimental validation of the larvicidal activity of the nanoemulsion.

## 4. Conclusions

Andiroba oil is known to have several biological activities and can provide innovative products for a wide range of industrial segments. However, to our knowledge, almost no efforts have previously been made to generate nanoemulsions of andiroba oil. Our study shows, for the first time, the preparation of these colloidal systems using a low-energy and solvent-free method. Characterization of the andiroba oil showed that it is basically a solution of limonoids and steroids dissolved in fatty acids. We also performed a simple classical larvicidal test using* Aedes aegypti *on a preliminary residual approach in order to verify the biological potential of the nanoemulsion. This choice was due to the fact that larvicidal activity of andiroba oil is well known and aiming to easily verify the potential bioactivity of the nanoemulsion. A potential controlled release of compounds may be involved, even if additional studies aiming to quantify the chemical markers and correlate them with this activity should be encouraged. Thus, the ecofriendly approach involved in the preparation associated with great bioactive potential of* C. guianensis *secondary metabolites makes this nanoemulsion very promising for valorization of this Amazon raw material that is obtained through a sustainable use of the rainforest.
